# Determination of Caffeine, Theobromine, and Theophylline by HPLC-DAD in Beverages Commonly Consumed in Lima, Peru

**DOI:** 10.1155/2023/4323645

**Published:** 2023-01-09

**Authors:** Karolinhe A. Gonzales-Yépez, Jorge Luis Vilela, Oscar Reátegui

**Affiliations:** ^1^Carrera de Nutrición y Dietética, Universidad Científica del Sur, Carretera Panamericana Sur km 19, Villa El Salvador, Lima 15058, Peru; ^2^Grupo de Investigación, Caracterización, Transformación y Sustentabilidad de los Recursos Naturales del Perú (CTS Group), Universidad Científica del Sur, Carretera Panamericana Sur km 19, Villa El Salvador, Lima 15058, Peru

## Abstract

The purpose of this study was to determine the content of caffeine, theobromine, and theophylline by high-performance liquid chromatography with diode-array detection (HPLC-DAD) in beverages commonly consumed in Lima, Peru. The samples were divided into 6 groups (herbal teas, coffee powder, chocolate milks, soft drinks, sports drinks, and energy drinks) which included the five most commonly consumed beverages of the different groups. Caffeine was mainly identified in the soft drink and energy drink groups, with the latter having a more significant value (10.38 ± 0.01 vs. 95.50 ± 3.48 mg/L, respectively). In herbal teas, caffeine showed the highest content (0.47 ± 0.01 to 4.91 ± 0.05 mg/L), despite theophylline being a characteristic compound of tea leaves. Sports drinks presented very low caffeine levels (0.03 ± 0.01 to 0.05 ± 0.01 mg/L), and theobromine (0.48 ± 0.01 to 6.00 ± 0.02) was also identified. Caffeine (4.09 ± 0.01 to 5.70 ± 0.01 mg/L) and theobromine (1.70 ± 0.01 to 12.24 ± 0.01 mg/L) were found in the five commercial brands of chocolate milk evaluated. Moreover, the group of coffee powder samples had the highest level of caffeine content (49.25 ± 0.24 to 964.40 ± 4.93 mg/100 g). The results obtained in this study provide reliable information on the composition and quantification of methylxanthines in the beverages most consumed in Lima and impact consumer knowledge.

## 1. Introduction

In the last decades, several studies have focused on the biologically active ingredients, particularly alkaloids in beverages, for their possible beneficial effects on human health [1]. One of those alkaloids is methylxanthine, a compound commonly present in food [2]. Monteiro et al. [3] noted that both animals and plants naturally produce these compounds, being caffeine, theobromine, and theophylline as the most frequently studied. These compounds are mainly found in food such as coffee beans, cocoa beans, and tea leaves [4].

These three methylxanthines are chemically very similar [5], and according to De Sena et al. [6], they share stimulating effects on the central nervous system as well as on other systems such as the gastrointestinal, cardiovascular, renal, and respiratory systems.

Caffeine, for example, is one of the best known and most studied substances, having a stimulating effect on the central nervous system, increasing alertness, and improving long-term memory and concentration and may also improve physical performance in athletes [7–9]. There is even a debate about the neuroprotective effects against certain types of degenerative diseases such as Alzheimer's and Parkinson's [10]. According to a Canadian study [11], moderate daily caffeine intake of up to 400 mg per day by healthy adults is not associated with adverse health effects. Likewise, the Food and Drug Administration (FDA) states that a dose of up to 400 mg of caffeine per day is safe in the healthy adult population, although this may vary according to the sensitivity of the person or vulnerable group (pregnant or breastfeeding women or individuals with any special health condition) [12]. However, excessive consumption may be related to hypertension, anxiety, hyperactivity, and headaches [7].

There are fewer studies on theobromine, which is present in high concentrations in cocoa [13]. According to Martínez-Pinilla et al. [13], the main mechanisms of action are inhibition of phosphodiesterases and blockade of adenosine receptors. Another clinical study suggests that theobromine could be the main active component of cocoa responsible for the effect of increasing HDL cholesterol [14]. However, there is no specific and/or conclusive information regarding their safe or lethal daily dose.

Theophylline has similar pharmacological and toxicological properties to those of caffeine [2]. According to Dolder [15], this compound can mainly be found in tea and in asthma medication used for bronchodilation. Although there is no precise information on a recommended dose, Greene et al. [16] have reported that inadequate consumption may cause toxic effects including tachycardia, hypertension, nausea, vomiting, and diarrhea.

In the last years, access to a huge variety of products [17], such as ready-to-drink beverages, coffee powders, soft drinks, energy drinks, sport drinks, and herbal teas, has become available. These products are commonly marketed in Peru, and most of the nutritional labeling does not clearly indicate the concentrations of certain compounds that they contain, with this information often remaining unknown to the consumer public.

Therefore, the aim of this study was to determine the content of caffeine, theobromine, and theophylline by high-performance liquid chromatography with diode-array detection (HPLC-DAD) and the antioxidant capacity in beverages commonly consumed in Lima, Peru.

## 2. Material and Methods

### 2.1. Chemicals

All the solvents and reagents were analytically graded with deionized water (Milli-Q quality) [4]. The standards of caffeine, theobromine, and theophylline were purchased from Sigma-Aldrich (Merck Peruana, Lima). Acetonitrile and methanol (HPLC grade) were also obtained from Sigma-Aldrich (Merck Peruana, Lima).

### 2.2. Sample Collection

The samples were divided into six groups: herbal teas (HT), sports drinks (SD), chocolate milk (CM), soft drinks (SOD), energy drinks (ED), and coffee powder (CP). Each group included five of the most popular beverages of the group, obtained by nonprobabilistic convenience sampling, based on a survey of consumers in Lima. Samples were purchased in three supermarkets in Metropolitan Lima during 2020. The nutritional labels of each sample analyzed were reviewed to corroborate the declared composition.

HTs are ready-to-drink cold drinks marketed as herbal infusions with an added value (e.g., fruit flavors or various properties), which are highly appreciated by a certain sector of the national and international markets [18]. SDs provide carbohydrates, electrolytes, and liquids to the body, helping the body hydrate before, during, and after physical activity [19].

CM is a mixture based on milk, sucrose, cocoa and/or cocoa powder, and some hydrocolloids, which are added to improve the consistency and avoid sedimentation of the cocoa particles [20].

On the other hand, SODs are flavored drinks, which are produced by adding CO_2_ gas directly to the drink, diluted with sucrose, and may or may not contain caffeine depending on the drink [21].

EDs are used to provide an extra burst of energy as well as promote wakefulness, increase attention span, maintain alertness, and improve athletic performance [22].

Lastly, CP is a drink prepared with coffee beans in powder form [23], with caffeine as its main compound. All coffee powder samples were prepared.

None of the samples in this study were sweetened with noncaloric sweeteners.

### 2.3. Total Polyphenol Measurement

The content of total phenolic was determined according to Singleton and Rossi [24]. Briefly, 1 mL of 10% Folin-Ciocalteu's reagent was mixed with 0.1 mL of sample for 5 min at room temperature; then, 1 mL of 5% sodium carbonate was added, and the mixture was placed in a water bath at 45°C for 15 min. All samples were analyzed in triplicate. Absorbance was read on a spectrophotometer at 725 nm. The results were expressed in mg gallic acid equivalent/100 mL (mg GAE/100 mL).

### 2.4. Total Flavonoid Measurement

Total flavonoid measurement was performed according to Wolfe and Liu [25]. In a 0.250 mL sample, 0.075 mL of 5% sodium nitrite was added and allowed to react for 5 min. Then, 0.150 mL of aluminum chloride was added, and the mixture was left to stand for 5 min. Finally, 0.275 mL of sodium hydroxide (1 M) was added to the mixture, and it was left to react for 15 min. All samples were analyzed in triplicate. The reading was performed at 510 nm in a spectrophotometer. The results were expressed in *μ*g catechin equivalent/100 mL (*μ*g CE/100 mL).

### 2.5. Antioxidant Capacity by Ferric Reducing Antioxidant Power

Antioxidant activity evaluated by the ferric reducing antioxidant power (FRAP) assay was performed according to the methodology proposed by Benzie and Strain [26]. Briefly, 1 mL of distilled water and 1 mL of the FRAP reagent were added to 0.1 mL of sample. The mixture was then placed in a 37°C water bath and allowed to react for 10 min. The reading was made in a spectrophotometer (Pharo 300, Spectroquant, USA) at 593 nm. A standard curve was prepared using different concentrations of Fe^2+^ ranging from 15 to 75 mM. All samples were analyzed in triplicate. The results were expressed in *μ*M Fe^2+^/100 mL (*μ*M Fe^2+^/100 mL).

### 2.6. Chromatographic Conditions

HPLC analyses of caffeine, theobromine, and theophylline were performed according to Srdjenovic et al. [4], with slight modifications. Briefly, a VWR HITACHI Chromaster 600 HPLC with a diode-array detector (DAD CM 5430), autosampler, and a reversed phase purospher STAR RP-8 column (5 *μ*m particle size, i.d. 4.6 × 150 mm) was used in an isocratic elution mode with the mobile phase water-THF(A) (0.1% THF in water, pH 8)-acetonitrile (B) (90 : 10, *v*/*v*). The pH was adjusted with 0.1 M NaOH. The mobile phase and all the solutions were filtered (0.45 *μ*m × 47 mm Millipore nylon filter), and the run time was 5 min, with a flow rate of 0.8 mL/min. The column temperature was 25°C, and the analytes were detected at 273 nm. The quantification of individual compounds was calculated with a calibration curve of the standard compound [27]. All samples were run in triplicate for each analysis.

### 2.7. Sample Preparation

The HTs and SDs were filtered through a 0.22 *μ*m nylon filter. Ten milliliters of the filtrate was adjusted to pH 8 with 0.1 M NaOH [4].

The CMs (25 mL) containing suspended particles were filled up to 200 mL with water in a separate container and extracted for 30 minutes at 60°C in an ultrasonic bath. The extracted sample was filtered through filter paper to remove solids. Then, 10 mL of the filtrate was adjusted to pH 8 with a 0.1 M NaOH solution [4].

The SOD and ED samples were degassed for 15 min to release CO_2_. Before analysis, the samples were adjusted to pH 8 with a 0.1 M NaOH solution and filtered through the 0.22 *μ*m × 33 mm Millipore Millex-GN nylon filter.

The CP samples were weighed (5 g) and extracted with boiling water (200 mL) and then mixed in a thermal flask for 5 min on the magnetic stirrer [4]. The extracts were then filtered, and 10 mL of the filtrate was adjusted to pH 8 with 0.1 M NaOH.

### 2.8. Cleanup Procedure

According to Srdjenovic et al. [4], the Supelclean LC-18 SPE cartridges were conditioned with 2 × 6 mL of methanol, followed by 2 × 6 mL of ultrapure deionized water. The extracts from each sample were then passed through the solid phase extraction (SPE) cartridges, followed by washing with 6 mL of ultrapure deionized water. They were air dried under vacuum for 10 min, and the fluids were discarded. Lastly, caffeine, theobromine, and theophylline were extracted from the SPE cartridges with 10 mL of chloroform. The solution was evaporated until dryness. The residue of all samples was reconstituted in 1 mL of pH 8 water, with the exception of CM, which was reconstituted in 2 mL. Samples were filtered through a 0.22 *μ*m nylon filter and injected into the HPLC [4].

### 2.9. Statistical Analysis

The results of each analysis were presented in tables, and the statistical analyses were performed using JASP version 0.14.1 (2020). Preliminarily, it was tested whether the variables met the assumptions of normality by means of the Shapiro-Wilk test, with a significance level of *p* > 0.05. In the comparison tests between beverage groups, the Kruskal-Wallis test was used, with a *p* value < 0.05 being considered significant.

## 3. Results and Discussion

### 3.1. Bioactive Compounds and Antioxidant Capacity

The total polyphenol content, total flavonoid content, and FRAP of the six groups of beverages are presented in Table 1. As can be seen, the total polyphenol content in the different beverages varied according to the group, with reductions in the following order: CP > ED > HT > CM > SOD > SD (Table 1). These differences are due to the numerous phenolic compounds found in coffee ranging from 3.57 ± 0.17 to 19.97 ± 0.17 mg GAE/100 mL compared to the other samples studied. In fact, chlorogenic acid attributes to more potential benefits of coffee and has shown to contribute to the prevention of Alzheimer's disease-induced cognitive dysfunction [28].

However, in the group of HTs, there was a notable difference in polyphenol content between the infusions based on black tea (HT-3, HT-4, and HT-5: 0.06 ± 0.01 to 0.40 ± 0.01 mg GAE/100 mL) and green tea (HT-1 and HT-2: 4.08 ± 0.27 to 4.74 ± 0.18 mg GAE/100 mL). In agreement with Horžić et al. [1], green tea was found to be the richest source of total phenols.

**Figure 1:**
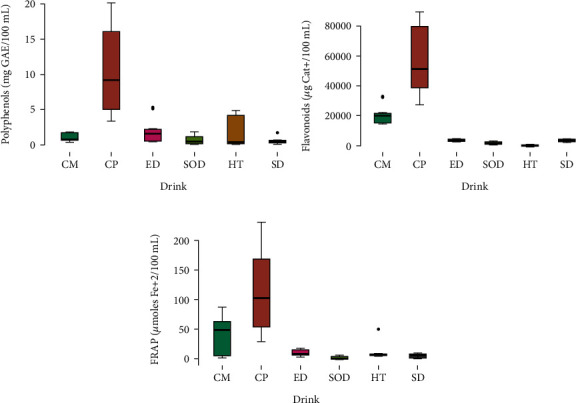
Box plot displaying the distribution of the concentrations of (a) polyphenols (mg GAE/100 mL), (b) flavonoids (*μ*g Cat+/100 mL), and (c) FRAP (*μ*moles Fe+2/100 mL) detected in the samples analyzed for the different brands of chocolate milks (CM), coffee powder (CP), energy drinks (ED), soft drinks (SOD), herbal teas (HT), and sport drinks (SD).

Regarding the total flavonoid content, CP and CMs presented the highest concentrations (57445.07 ± 5969.47 *μ*g CE(+)/100 mL and 20785.92 ± 7315.11 *μ*g CE(+)/100 mL, respectively) compared to the other groups (Figure 1(a)).

According to the results obtained, the group with the lowest flavonoid content was the HTs (Figure 1(b)). No previous study has compared these groups of beverages to establish why teas have lower flavonoid content. On the other hand, among the tea beverages, it is conceivable that the catechin content may vary according to tea variety, origin, time of harvest, and sun exposure [1], and these factors should be considered in future research. The FRAP assay showed that CP had a much higher antioxidant capacity (29.51 ± 0.29 to 228.88 ± 1.60 *μ*mol Fe(2+)/100 mL) compared to the other samples, such as SOD, which showed a very low or almost no antioxidant capacity (Figure 1(c)).

Based on these results, the group of CP drinks predominated in most of the tests carried out, presenting the greatest antioxidant capacity (Figure 1(c)) due to its bioactive compounds such as chlorogenic acid. The other groups varied in relation to the compounds of each sample.

### 3.2. Results of Methylxanthine (CF, TB, and TP) Measurement

**Figure 2:**
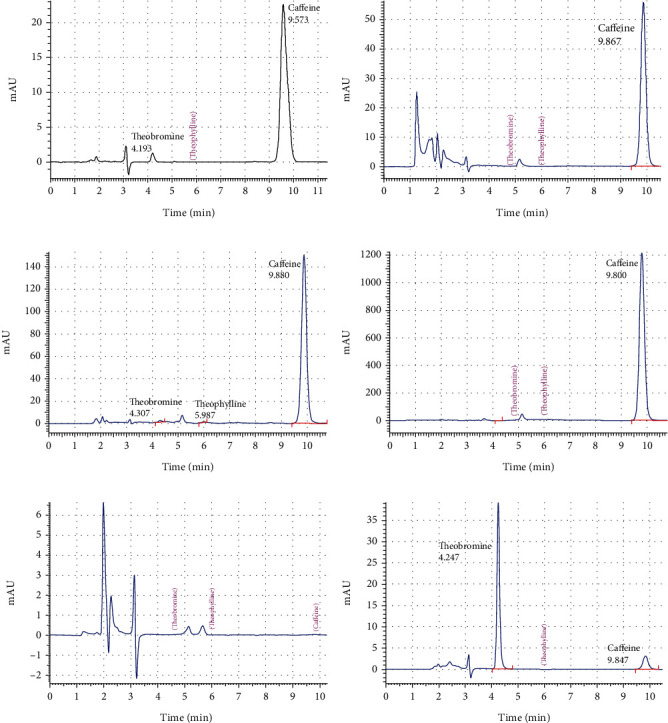
Chromatograms of some samples by HPLC-DAD: (a) HT-3, (b) SOD-5, (c) CP-1, (d) ED-2, (e) SD-4, and (f) CM-5.

The content of caffeine, theobromine, and theophylline obtained by the HPLC-DAD method in the different beverage samples is shown in Table 2 and Figure 2. The declared and determined values of these methylxanthines found by HPLC-DAD in the analyses of the beverages are also shown.

#### 3.2.1. Soft Drinks and Energy Drinks

**Figure 3:**
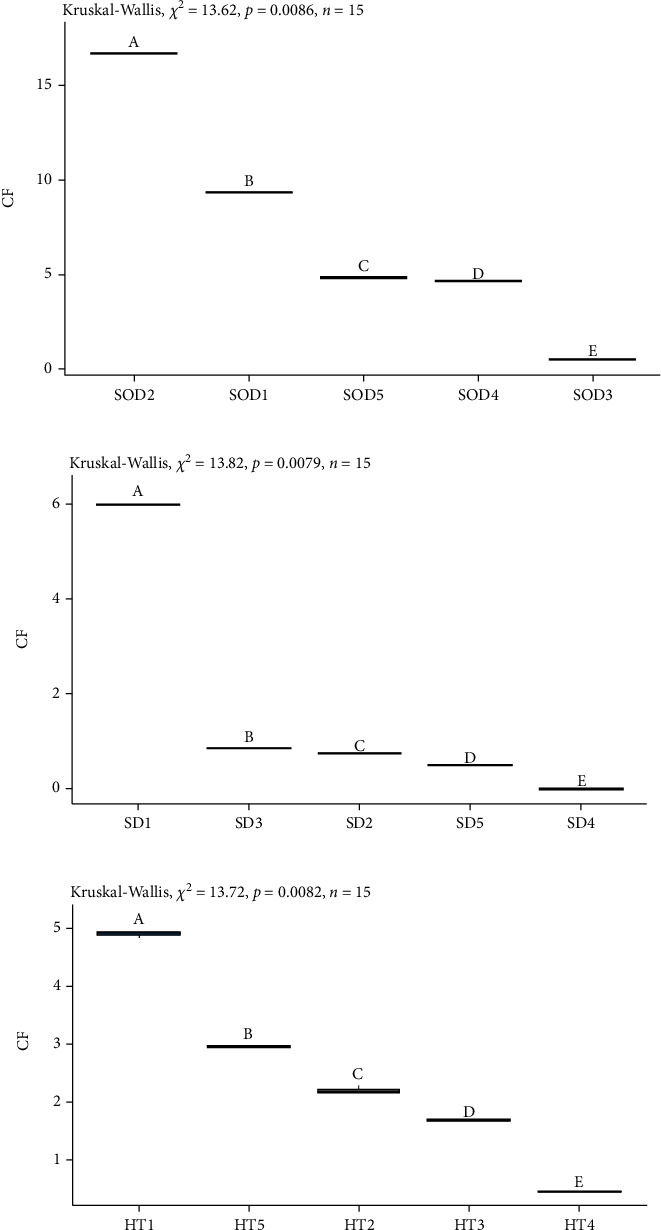
Box plot showing the distribution of the concentrations of the most representative methylxanthine in each group: (a) soft drink-caffeine (mg/100 g sample), (b) sport drink-caffeine (mg/100 g sample), and (c) herbal tea-caffeine (mg/100 g sample).

**Figure 4:**
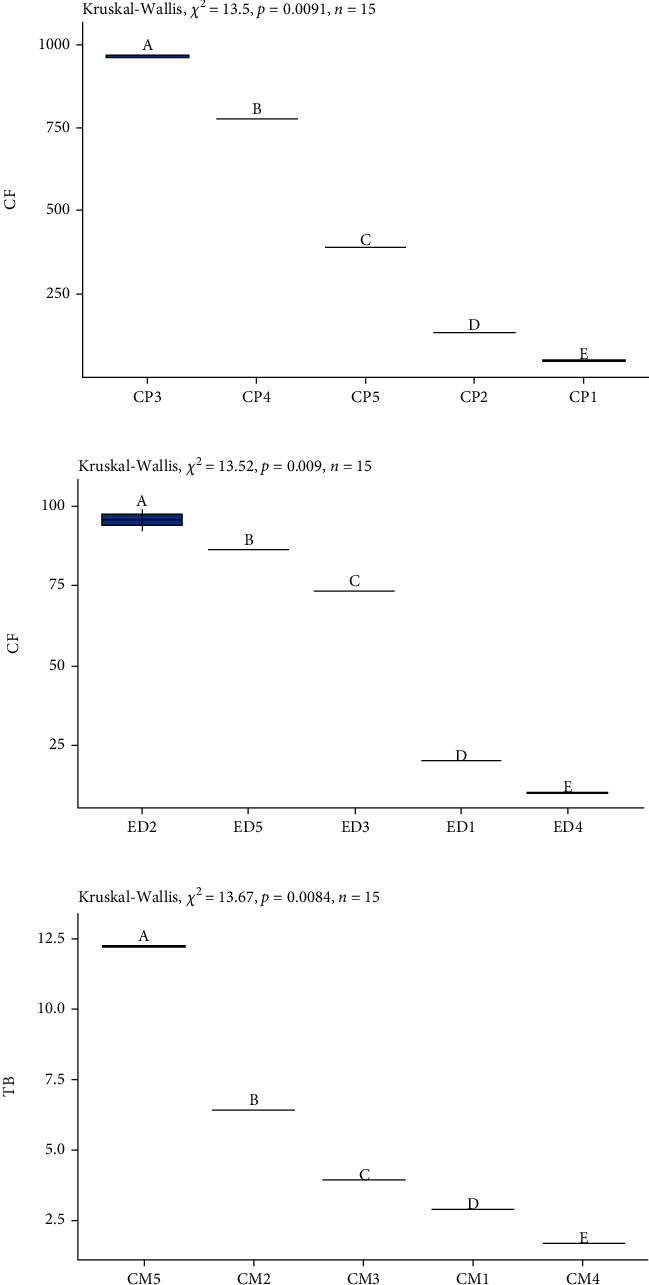
Box plot showing the distribution of the concentrations of the most representative methylxanthine in each group: (a) coffee powder-caffeine, (b) energy drink-caffeine, and (c) chocolate milk-theobromine.

The SOD and ED groups had no detectable amounts of theobromine or theophylline. These groups were characterized by caffeine as the most predominant methylxanthine. The content of caffeine in SOD ranged from 0.54 ± 0.01 mg/L for SOD-3 to 16.68 ± 0.01 mg/L for SOD-2 (Figure 3(a)). The caffeine content of EDs ranged from 10.38 ± 0.01 for ED-4 to 95.5 ± 3.48 mg/L for ED-2 (Figure 4(b)), being almost 8-fold greater than the SODs analyzed.

In comparison with Serbia [4], one study reported that caffeine content in EDs ranged from 237 mg/L to 348.7 mg/L, with a 2- to 3-fold greater caffeine content than SOD with a minimum value of 106.7 mg/L and a maximum of 119.1 mg/L. Therefore, while the ratio of caffeine content between EDs and SODs seems to be higher in this study, their values were lower.

According to several studies [29], caffeine is one of the most widely used psychoactive substances in the world and is often used as an additive in SOD [30] and ED [31].

On the other hand, most of the commercial brands analyzed in these categories do not declare the total caffeine content of their product. However, it is included in the list of ingredients on the nutrition label.

**Figure 5:**
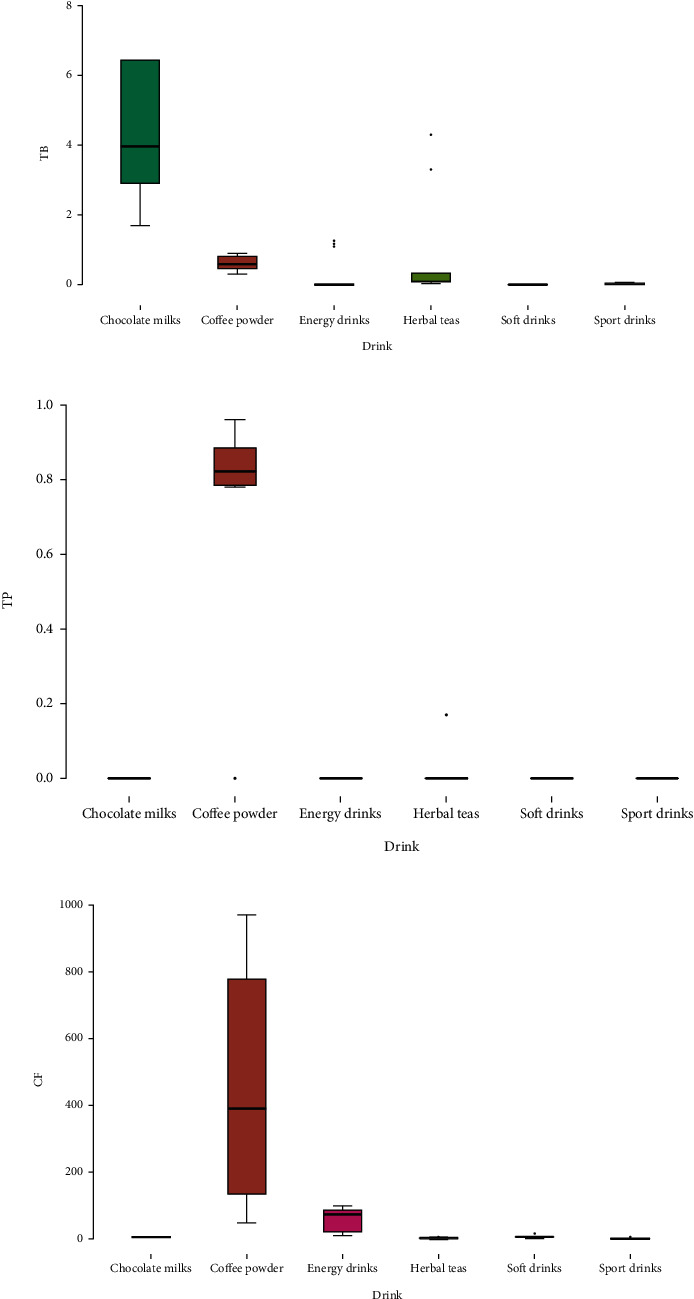
Box plot showing the distribution of the concentrations of methylxanthines: (a) theobromine (mg/L), (b) theophylline (mg/L), and (c) caffeine (mg/100 g sample).

In the SOD and ED groups, the amount of total caffeine content was below the lower limit of consumption of 400 mg per day in healthy adults [11, 12]. However, it was of note that ED had the second highest caffeine content (Figure 5(c)). Only two brands of the five EDs indicated the amount of caffeine contained by their product in the nutritional labeling. It is important for this information to be included on the nutritional labeling of the product in order for consumers to control their consumption.

#### 3.2.2. Herbal Teas and Sport Drinks

The groups of HT and SD showed the presence of only two methylxanthines, theobromine and caffeine. In the HT, the highest content identified was caffeine (0.47 ± 0.01 to 4.91 ± 0.05 mg/L), with the lowest value in HT-4 and the highest value in HT-1, respectively (Figure 3(c)). One would expect to find theophylline in the HTs as it is a characteristic and predominant compound in tea leaves [16], and some are marketed precisely because of the beneficial health properties of tea leaves.

However, according to De Paula and Farah [32], there is no perceptible trace of this methylxanthine in these HTs possibly due to the fact that these infusion drinks are sweetened, contributing to the dilution of the tea solids and producing a higher content of soluble solids. In addition, many other factors could intervene in the variation of the properties of the product.

The results obtained are consistent with other studies [4, 33, 34], which evaluated different beverages and described that ready-to-drink HTs have a high content of caffeine and also of theobromine in some samples.

The composition of SDs is basically carbohydrates and electrolytes [19], and their nutritional labeling does not indicate or allude to any ingredient containing caffeine, theobromine, or theophylline, and therefore, they should not present significant values of these methylxanthines. According to the results obtained, almost undetectable values of only two methylxanthines, caffeine (0.03 ± 0.01 to 0.05 ± 0.01 mg/L) and theobromine (0.48 ± 0.01 to 6.00 ± 0.02 mg/L), were found in the SDs analyzed (Table 2 and Figure 3(b)), thereby confirming and validating the expected content.

#### 3.2.3. Chocolate Milks (CM)

The CM drinks are mixture based on milk, sucrose, and cocoa and/or cocoa powder [21], cocoa being the ingredient leading to the denomination of a CM. According to some studies [35], cocoa is rich in two methylxanthines, predominantly theobromine followed by caffeine.

Among the five commercial brands of chocolate milk evaluated (Figure 5(a)), theobromine was identified in greater quantity (1.7 ± 0.01 mg/L to 12.24 ± 0.01 mg/L) followed by caffeine (4.09 ± 0.01 mg/L to 5.69 ± 0.01 mg/L). According to two studies, one carried out in Serbia [4] and the other in Spain [36], the mean theobromine content of the CM evaluated was 225.8 mg/L and 205.7 mg/L, while the mean caffeine content was 5.6 mg/L and 18.4 mg/L, respectively. However, the values found in the Serbian and Spanish studies were much higher than those found in the beverages analyzed in the present study.

On the other hand, Table 2 shows that theobromine levels varied greatly between one of the brands of CM (CM-5) compared to the others analyzed (Figure 4(c)). The CM-5 sample had two to four times more theobromine than the other brands of CM (Figure 4(c)). According to its nutritional labelling, the CM-5 brand of CM included cocoa in second place in its list of ingredients, while in the other brands, cocoa appears as the third ingredient. This would explain the difference in the concentration of theobromine in the CM-5 drink (Table 2).

Taking into account these differences in theobromine concentrations in the different CM products, it is important to understand and know the content of each compound included in the products consumed.

### 3.3. Coffee Powder (CP)

Among the six beverage groups evaluated, CP had the highest level of caffeine in CP-3 (49.25 ± 0.24 mg/100 g) and the lowest value in CP-1 (964.40 ± 4.93 mg/100 g) (Figure 4(a)).

The coffee bean is the main source of caffeine [22] and the main ingredient of the samples evaluated, which corroborates the results found. However, the level of caffeine in each of the five samples studied varied, likely due to the variety of coffee bean used by each brand [36], among other factors, for the processing treatment of this product.

As can be seen, compared to the other CP samples, two (CP-3 and CP-4) presented the highest level of caffeine (964.40 ± 4.93 and 778.19 ± 0.61 mg/100 g sample, respectively) (Figure 4(a)). This means that, in the case of CP-3, a person would be consuming approximately 19.29 mg of caffeine per cup, provided that only 2 g of coffee powder had been added, as suggested in the nutritional labels of the product. This does not exceed the limit described in several studies [11, 12], reporting that an intake of up to 400 mg/day is not associated with adverse health effects.

While some people choose to consume this drink for its characteristics, benefits, or simply its taste, it must be remembered that excess intake is related to negative effects such as hypertension, insomnia, constant agitation, headaches, and anxiety [7].

Furthermore, people sensitive to caffeine or nonregular consumers or those with a health problem in which caffeine interferes or aggravates their symptoms [32] should take greater care and should be taken to limit or restrict their consumption.

## 4. Conclusions

The results obtained in this study provide reliable information on the composition and quantification of methylxanthines in the beverages most consumed in Lima, using the HPLC-DAD method.

The group of CPs predominated as having the highest antioxidant activity according to the tests carried out. Similarly, this group presented the highest level of caffeine, which was to be expected since its main active ingredient is the coffee bean.

Theobromine was predominantly found in the group of CMs analyzed.

Although theophylline is mainly found in the tea leaf, there was no detectable or significant value within the group of ready-to-drink HTs, and little antioxidant activity was identified. This should be analyzed in future research.

In general, it was observed that caffeine was one of the bioactive compounds present in each of the groups of beverages analyzed.

Inclusion of the concentrations of the different compounds found in the nutritional labels of food products is essential. This information should be stated in a clear and understandable way to consumers, improving the nutritional education of the population and favoring purchasing decisions.

## Figures and Tables

**Table 1:** Total polyphenols, flavonoids, and FRAP results of the beverages most consumed in Lima, Peru^∗^.

Beverage Samples	Total polyphenols (mg GAE/100 mL)	Total flavonoids (*μ*g CE(+)/100 mL)	FRAP (*μ*mol Fe^2+^/100 mL)
SOD-1	1.12 ± 0.04	2718.31 ± 70.42	3.59 ± 0.10
SOD-2	0.19 ± 0.01	915.49 ± 70.42	<LOD
SOD-3	0.08 ± 0.01	887.32 ± 42.25	<LOD
SOD-4	0.46 ± 0.04	971.83 ± 42.25	<LOD
SOD-5	1.85 ± 0.01	2647.89 ± 28.17	6.17 ± 0.15
ED-1	1.58 ± 0.02	3732.39 ± 42.25	15.63 ± 0.10
ED-2	5.19 ± 0.10	4126.76 ± 70.42	17.04 ± 0.05
ED-3	0.57 ± 0.10	2723.00 ± 70.89	6.21 ± 0.29
ED-4	2.16 ± 0.04	3859.15 ± 28.17	8.58 ± 0.15
ED-5	0.49 ± 0.02	2971.83 ± 79.42	3.06 ± 0.05
HT-1	4.74 ± 0.18	4.00 ± 0.40	6.55 ± 0.05
HT-2	4.08 ± 0.27	7.33 ± 0.61	38.98 ± 0.05
HT-3	0.40 ± 0.01	9.20 ± 0.40	7.91 ± 0.05
HT-4	0.06 ± 0.01	<LOD	5.08 ± 0.06
HT-5	0.12 ± 0.03	37.40 ± 0.60	4.85 ± 0.10
SD-1	0.53 ± 0.03	2873.24 ± 56.34	0.74 ± 0.06
SD-2	0.11 ± 0.02	3070.42 ± 84.51	<LOD
SD-3	0.58 ± 0.06	4225.35 ± 56.34	2.23 ± 0.10
SD-4	0.38 ± 0.01	3436.62 ± 56.34	7.43 ± 0.05
SD-5	1.76 ± 0.05	2323.94 ± 79.42	9.66 ± 0.05
CM-1	0.38 ± 0.01	21647.89 ± 211.27	86.60 ± 0.42
CM-2	0.74 ± 0.02	14859.15 ± 14.08	47.91 ± 0.34
CM-3	0.76 ± 0.28	14774.65 ± 211.27	4.95 ± 0.10
CM-4	1.80 ± 0.03	32676.06 ± 281.69	62.77 ± 0.53
CM-5	1.69 ± 0.03	19971.83 ± 169.01	1.41 ± 0.05
CP-1	3.57 ± 0.17	51154.93 ± 225.35	29.51 ± 0.29
CP-2	5.09 ± 0.16	78971.83 ± 1478.87	54.08 ± 0.39
CP-3	15.92 ± 0.24	89478.87 ± 70.42	167.86 ± 0.49
CP-4	19.97 ± 0.17	38873.24 ± 647.89	228.88 ± 1.60
CP-5	9.23 ± 0.28	28746.48 ± 1450.70	103.20 ± 0.97

Note^∗^: LOD: level of detail; SOD: soft drinks; ED: energy drinks; HT: herbal teas; SD: sport drinks; CM: chocolate milks; CP: coffee powder; CE: catechin equivalent.

**Table 2:** HPLC-DAD determination of theobromine, theophylline, and caffeine concentrations in the beverage most frequently consumed in Lima^∗^.

Beverage samples	Values found			Declared values		
	TB	TP	CF	TB	TP	CF
SOD-1 (mg/L)	<LOD	<LOD	9.34 ± 0.02	n.d	n.d	n.d
SOD-2 (mg/L)	<LOD	<LOD	16.68 ± 0.01	n.d	n.d	n.d
SOD-3 (mg/L)	<LOD	<LOD	0.54 ± 0.01	n.d	n.d	n.d
SOD-4 (mg/L)	<LOD	<LOD	4.63 ± 0.02	n.d	n.d	n.d
SOD-5 (mg/L)	<LOD	<LOD	4.82 ± 0.06	n.d	n.d	n.d
ED-1 (mg/L)	1.17 ± 0.09	<LOD	20.38 ± 0.12	n.d	n.d	n.d
ED-2 (mg/L)	<LOD	<LOD	95.50 ± 3.48	n.d	n.d	≤320
ED-3 (mg/L)	<LOD	<LOD	73.45 ± 0.02	n.d	n.d	n.d
ED-4 (mg/L)	<LOD	<LOD	10.38 ± 0.01	n.d	n.d	≤200
ED-5 (mg/L)	<LOD	<LOD	86.58 ± 0.04	n.d	n.d	n.d
HT-1 (mg/L)	0.31 ± 0.01	0.17 ± 0.01	4.91 ± 0.05	n.d	n.d	n.d
HT-2 (mg/L)	3.64 ± 0.58	<LOD	2.21 ± 0.08	n.d	n.d	n.d
HT-3 (mg/L)	0.07 ± 0.01	<LOD	1.70 ± 0.01	n.d	n.d	n.d
HT-4 (mg/L)	0.03 ± 0.01	<LOD	0.47 ± 0.01	n.d	n.d	n.d
HT-5 (mg/L)	0.09 ± 0.01	<LOD	2.96 ± 0.01	n.d	n.d	n.d
SD-1 (mg/L)	0.05 ± 0.01	<LOD	6.00 ± 0.02	n.d	n.d	n.d
SD-2 (mg/L)	0.03 ± 0.01	<LOD	0.75 ± 0.01	n.d	n.d	n.d
SD-3 (mg/L)	0.04 ± 0.01	<LOD	0.84 ± 0.01	n.d	n.d	n.d
SD-4 (mg/L)	<LOD	<LOD	<LOD	n.d	n.d	n.d
SD-5 (mg/L)	<LOD	<LOD	0.48 ± 0.01	n.d	n.d	n.d
CM-1 (mg/L)	2.92 ± 0.01	<LOD	4.71 ± 0.01	n.d	n.d	n.d
CM-2 (mg/L)	6.43 ± 0.01	<LOD	5.70 ± 0.01	n.d	n.d	n.d
CM-3 (mg/L)	3.96 ± 0.01	<LOD	5.37 ± 0.01	n.d	n.d	n.d
CM-4 (mg/L)	1.70 ± 0.01	<LOD	4.09 ± 0.01	n.d	n.d	n.d
CM-5 (mg/L)	12.24 ± 0.01	<LOD	5.69 ± 0.01	n.d	n.d	n.d
CP-1 (mg/100 g sample)	0.45 ± 0.01	0.79 ± 0.01	49.25 ± 0.24	n.d	n.d	n.d
CP-2 (mg/100 g sample)	0.30 ± 0.01	0.95 ± 0.01	133.00 ± 0.07	n.d	n.d	n.d
CP-3 (mg/100 g sample)	0.83 ± 0.07	0.87 ± 0.01	964.40 ± 4.93	n.d	n.d	n.d
CP-4 (mg/100 g sample)	0.80 ± 0.01	0.87 ± 0.03	778.19 ± 0.61	n.d	n.d	n.d
CP-5 (mg/100 g sample)	0.58 ± 0.02	<LOD	389.79 ± 0.13	n.d	n.d	n.d

Note^∗^: TB: theobromine; TP: theophylline; CF: caffeine; n.d: not declared; LOD: level of detail; SOD: soft drinks; ED: energy drinks; HT: herbal teas; SD: sport drinks; CM: chocolate milks; CP: coffee powder.

## Data Availability

The datasets generated during and/or analyzed during the current study are available from the corresponding author on reasonable request.

## References

[1] Horžić D., Komes D., Belščak A., Kovačević K., Iveković D., Karlović D. (2009). The composition of polyphenols and methylxanthines in teas and herbal infusions. *Food Chemistry*.

[2] Martínez-López S., Sarriá B., Gómez-Juaristi M., Goya L., Mateos R., Bravo-Clemente L. (2014). Theobromine, caffeine, and theophylline metabolites in human plasma and urine after consumption of soluble cocoa products with different methylxanthine contents. *Food Research International*.

[3] Monteiro J., Alves M., Oliveira P., Silva B. (2016). Structure-bioactivity relationships of methylxanthines: trying to make sense of all the promises and the drawbacks. *Molecules*.

[4] Srdjenovic B., Djordjevic-Milic V., Grujic N., Injac R., Lepojevic Z. (2008). Simultaneous HPLC determination of caffeine, theobromine, and theophylline in food, drinks, and herbal products. *Journal of Chromatographic Science*.

[5] Rodriguez A., Costa-Bauza A., Saez-Torres C., Rodrigo D., Grases F. (2015). HPLC method for urinary theobromine determination: effect of consumption of cocoa products on theobromine urinary excretion in children. *Clinical Biochemistry*.

[6] De Sena A., De Assis S., Branco A. (2011). Analysis of theobromine and related compounds by reversed phase high-performance liquid chromatography with ultraviolet detection: an update (1992-2011). *Food Technology and Biotechnology*.

[7] Gonzalez E., Vinicio M. (2014). Impact of caffeine and coffee on our health. *Trends in Endocrinology and Metabolism*.

[8] Olas B., Bryś M. (2019). Effects of coffee, energy drinks and their components on hemostasis: the hypothetical mechanisms of their action. *Food and Chemical Toxicology*.

[9] Barreda-Abascal R., Molina L., Haro-Valencia R., Alford C., Verster J. (2012). Actualización sobre los efectos de la cafeína y su perfil de seguridad en alimentos y bebidas. *Revista Médica del Hospital General de México*.

[10] Ikram M., Park T., Ali T., Kim M. (2020). Antioxidant and neuroprotective effects of caffeine against Alzheimer’s and Parkinson’s disease: insight into the role of Nrf-2 and A2AR signaling. *Antioxidants*.

[11] Nawrot P., Jordan S., Eastwood J., Rotstein J., Hugenholtz A., Feeley M. (2003). Effects of caffeine on human health. *Food Additives and Contaminants*.

[12] (2018). *FDA: How Much Caffeine Is Too Much?*.

[13] Martínez-Pinilla E., Oñatibia-Astibia A., Franco R. (2015). The relevance of theobromine for the beneficial effects of cocoa consumption. *Frontiers in Pharmacology*.

[14] Neufingerl N., Zebregs Y., Schuring E., Trautwein E. (2013). Effect of cocoa and theobromine consumption on serum HDL-cholesterol concentrations: a randomized controlled trial. *The American Journal of Clinical Nutrition*.

[15] Dolder L. K., Peterson M., Talcott P. (2013). Methylxanthines: caffeine, theobromine, theophylline. *Small Animal Toxicology*.

[16] Greene S. C., Halmer T., Carey J. M., Rissmiller B. J., Musick M. A. (2018). Theophylline toxicity: an old poisoning for a new generation of physicians. *Turkish Journal of Emergency Medicine*.

[17] Zegler J. (2018). Tendencias mundiales en alimentos y bebidas para 2018. https://www.siicex.gob.pe/siicex/documentosportal/alertas/documento/doc/277698773rad83597.pdf.

[18] Román A. (2003). *Infusiones Heladas como Bebidas Alternativas en el Mercado Nacional*.

[19] Simulescu V., Ilia G., Macarie L., Merghes P. (2019). Utilisation des boissons pour le sport et des boissons energisantes pendant et apres entrainement. *Science & Sport*.

[20] Yanes M., Durán L., Costell E. (2002). Rheological and optical properties of commercial chocolate milk beverages. *Journal of Food Engineering*.

[21] Ashurst P. R. (2016). Carbonated beverages. *Reference module in food science*.

[22] Scalese M., Denoth F., Siciliano V. (2017). Energy drink and alcohol mixed energy drink use among high school adolescents: association with risk taking behavior, social characteristics. *Addictive Behaviors*.

[23] López E., Giner R. (2013). Chocolate, café, té y otros estimulantes: bebidas energéticas avant la lettre (I). *Revista Española de Drogodependencias*.

[24] Singleton V., Rossi J. (1965). Colorimetry of total phenolics with phosphomolybdic-phosphotungstic acid reagents. *American Journal of Enology and Viticulture*.

[25] Wolfe K., Liu R. (2008). Structure-activity relationships of flavonoids in the cellular antioxidant activity assay. *Journal of Agricultural and Food Chemistry*.

[26] Benzie I., Strain J. (1996). The ferric reducing ability of plasma (FRAP) as a measure of “antioxidant power”: the FRAP assay. *Analytical Biochemistry*.

[27] Sen K., Sonmezdag A. S. (2020). Elucidation of phenolic profiling of cv. Antep Karasi grapes using LC-DAD-ESI-MS/MS. *Journal of Raw Materials to Processed Foods*.

[28] Ishida K., Yamamoto M., Misawa K. (2020). Coffee polyphenols prevent cognitive dysfunction and suppress amyloid *β* plaques in APP/PS2 transgenic mouse. *Neuroscience Research*.

[29] Ramirez C., Osorio J. (2013). Using caffeine for physical exercise: advantages and risks. *Revista de la Facultad de Medicina*.

[30] Keast R., Riddell L. (2007). Caffeine as a flavor additive in soft-drinks. *Appetite*.

[31] McCusker R., Goldberger B., Cone E. (2006). Caffeine content of energy drinks, carbonated sodas, and other beverages. *Journal of Analytical Toxicology*.

[32] De Paula L. J., Farah A. (2019). Methylxanthines in stimulant foods and beverages commonly consumed in Brazil. *Journal of Food Composition and Analysis*.

[33] Nazri M., Abdul A., Hayati N. (2015). *Quantification of Methylxanthines (Theobromine, Theophylline and Caffeine) in Chocolate, Tea and Coffee-Based Beverages by HPLC*.

[34] Al-Obaidi H., Afzal M. (2010). Methylxanthine content in hot drinks consumed in the State of Kuwait. *Journal of Food, Agriculture & Environment*.

[35] Langer S., Marshall L., Day A., Morgan M. (2011). Flavanols and methylxanthines in commercially available dark chocolate: a study of the correlation with nonfat cocoa solids. *Journal of Agricultural and Food Chemistry*.

[36] Sanchez J. (2017). Methylxanthine content in commonly consumed foods in Spain and determination of its intake during consumption. *Food*.

